# Next Generation Cancer Vaccines—Make It Personal!

**DOI:** 10.3390/vaccines6030052

**Published:** 2018-08-09

**Authors:** Angelika Terbuch, Juanita Lopez

**Affiliations:** 1 Drug Development Unit, Royal Marsden Hospital and The Institute of Cancer Research, Sutton SM2 5PT, UK; angelika.terbuch@icr.ac.uk or angelika.terbuch@medunigraz.at; 2 Division of Clinical Oncology, Department of Internal Medicine, Medical University of Graz, 8010 Graz, Austria

**Keywords:** cancer vaccines, immunotherapy, combination strategies, cancer immunity cycle, neoantigens, personalized cancer vaccine

## Abstract

Dramatic success in cancer immunotherapy has been achieved over the last decade with the introduction of checkpoint inhibitors, leading to response rates higher than with chemotherapy in certain cancer types. These responses are often restricted to cancers that have a high mutational burden and show pre-existing T-cell infiltrates. Despite extensive efforts, therapeutic vaccines have been mostly unsuccessful in the clinic. With the introduction of next generation sequencing, the identification of individual mutations is possible, enabling the production of personalized cancer vaccines. Combining immune check point inhibitors to overcome the immunosuppressive microenvironment and personalized cancer vaccines for directing the host immune system against the chosen antigens might be a promising treatment strategy.

## 1. Introduction

An effective host immune response against cancer depends on an intact chain of carefully regulated steps known as the cancer-immunity cycle. The first event is the release of cancer antigens, followed by antigen presentation to T cells on dendritic cells via major histocompatibility complex (MHC) class I and II molecules. This leads to priming and activation of T cells, followed by trafficking and infiltration of T cells into the cancer. Recognition of target cancer cells by T cells and destruction of the cancer are the final steps of the chain which leads to release of additional cancer antigens and subsequently restart of the cancer immunity cycle [1]. Cancer cells use different strategies to interfere with every step of the cancer immunity cycle including the activation of negative immunoregulatory pathways and upregulation of the immunosuppressive checkpoint programmed death-ligand 1 (PD-L1) [2,3,4,5,6]. Furthermore, cancer cells create their own immunosuppressive tumour microenvironment (TME). They secrete a variety of chemokines and inflammatory mediators like chemokine ligand 2 (CCL2), interleukin (IL) 6 and 10, transforming growth factor (TGF) ß, granulocyte-macrophage colony-stimulating factor (GM-CSF) and vascular endothelial growth factor (VEGF) that lead to recruitment of myeloid-derived suppressor cells (MDSCs), tumour-associated macrophages (TAMs) and tumour-associated neutrophils (TANs) [7]. MDSCs promote tumour invasion and metastases by secretion of soluble factors such as multiple matrix metalloproteinases (MMPs), VEGF, TGF-ß and S100A8/9, but they also play a key role in checkpoint regulation. Cancer associated hypoxia increases the expression of PD-L1 on MDSCs via induction of hypoxia-inducible factor 1-α [8]. TAMs and TANs also take part in checkpoint regulation. They produce cytokines and chemokines like TGF-ß and IL-10 to recruit T-regulatory cells (Tregs) into the TME [9]. Tregs are a subclass of CD4^+^ T cells, further classified by CD25 and FoxP3 expression, and are highly immunosuppressive. They express the checkpoint cytotoxic T-lymphocyte-associated protein 4 (CTLA-4) which binds to B7 molecules on antigen presenting cells (APCs) and thereby hinders the interaction of CD28 molecules on T cells and the B7 molecules on APCs [10].

The avoidance of immune destruction is now recognised as one of the hallmarks of cancer [11]. Monoclonal antibodies to these checkpoint proteins have shown clinical efficacy in a wide range of malignancies with approvals by the FDA for the treatment of a rapidly growing list of tumour types. Emerging translational analyses from the initial checkpoint inhibitor studies across tumour types have consistently demonstrated that checkpoint inhibitor therapies are most efficient in patients with pre-existing tumour infiltrating T-cells that were inhibited by PD-L1 [12,13]. In stark contrast, these therapies are much less efficacious in tumours devoid of infiltrating lymphocytes, suggesting that failure of effective T cell priming may hinder the generation of an effective immune response [14].

In this review, we aim to provide an overview of the current understanding of the dynamic interaction between tumour and host immune system, focusing on the key steps required for efficient T cell priming and how these can be subverted by cancer. We then discuss strategies to amplify tumour-specific T cell responses through therapeutic active immunization with vaccines, and in combination with checkpoint inhibitors. 

## 2. Tumour Antigens

Much work has gone into identifying tumour antigens able to drive effective T-cell responses against cancer (Table 1). In general, tumour antigens can be categorized as being tumour associated or tumour specific [15,16].

### 2.1. Tumour Associated Antigens

Tumour associated antigens are a group of non-mutant molecules that are “shared” by normal tissue and the cancer like overexpressed antigens or differentiation antigens. Amplification of genes like in Her2/Neu positive breast cancer can lead to overexpression of a normal protein. Differentiation antigens are molecules expressed on the cancer and on non-malignant cells of the same cell lineage, for example tyrosinase in melanoma cells which is also expressed in normal melanocytes. Since tumour associated antigens are self-proteins, they are more likely to have induced some form of centrally or peripherally mediated immunological tolerance often resulting in a reduced repertoire of immune effectors specific for the tumour associated antigen [16,17,18]. A potential problem in activating an immune response against differentiation antigens is the induction of autoimmune response against the normal tissue. Cancer–germline antigens are subsets of tumour associated antigens that are thought to provide higher tumour specificity as they are not expressed in normal adult tissues, except in germline and trophoblastic cells but are highly expressed across cancer. They include melanoma-associated antigen (MAGE)-A1, MAGE-A3, G antigen (GAGE), B-melanoma antigen (BAGE), and cancer testis antigen (NY-ESO-1) [15]. Other onco-fetal antigens, such as trophoblast glycoprotein (TPBG) are also thought to be specific to tumours as they are present only during fetal development [16,19]. However, all of these antigens are subject to a degree of tolerance and also lack complete specificity to tumour, leading to concerns about toxicity as seen in the recent trials with MAGE-A3 adoptive T-cells where cross-reaction with MAGE-A12 present in the brain led to neurotoxicity [15,16]. 

### 2.2. Tumour Specific Antigens

Tumour specific antigens are exclusively expressed by the cancer, and as such not subject to central tolerance, and are ideally suited as therapeutic targets. Oncogenic viral antigens can be used as targets in virus-associated cancers such as human papillomavirus (HPV) in cervical, anogenital and oropharyngeal cancers, hepatitis B virus in hepatocellular carcinoma and human herpesvirus 8 in Kaposi sarcoma [20,21]. Cytomegalovirus proteins are expressed in the majority of glioblastoma patients although their role in etiology is unclear [22,23]. Vaccines against oncogenic HPV infections are already FDA approved in the prophylactic setting but have also shown efficacy in the therapeutic setting [22]. However, only a minority of cancers are caused by viruses.

*Tumour neoantigens* arise from a somatic mutation in the cancer and can be common recurrent mutations such as the BRAF V600E mutation or Kirsten rat sarcoma (KRAS) G12D [24]. However, the majority of these shared mutant antigens are poorly immunogenic [16] and with the advance of more widely available next-generation sequencing techniques, it is now clear that tumours express a varied number of neoantigens [25,26]. Higher neoantigen load is associated with increased T-cell infiltration and improved outcomes [27,28,29]. Importantly, only a small fraction of putative mutated peptides are presented on MHC class I and/or MHC class II, and an even smaller subset of those are immunogenic [30,31]. The beauty of these neoantigens is that they are unique to the individual patient, and pave the way for personalized treatment strategies. 

Table 1 HER human epidermal growth factor receptor, TERT telomerase reverse transcriptase, PSA prostate specific antigen, MAGE melanoma-associated antigen, BAGE B-melanoma antigen, GAGE G antigen, NY-ESO-1 known as cancer testis antigen, CEA carcinoembryonic antigen, MUC mucin, TPBG trophoblast glycoprotein, HPV human papillomavirus, HBV hepatitis B virus, HHV human herpesvirus, KRAS Kirsten rat sarcoma

## 3. Antigen Presentation

**Figure 1:**
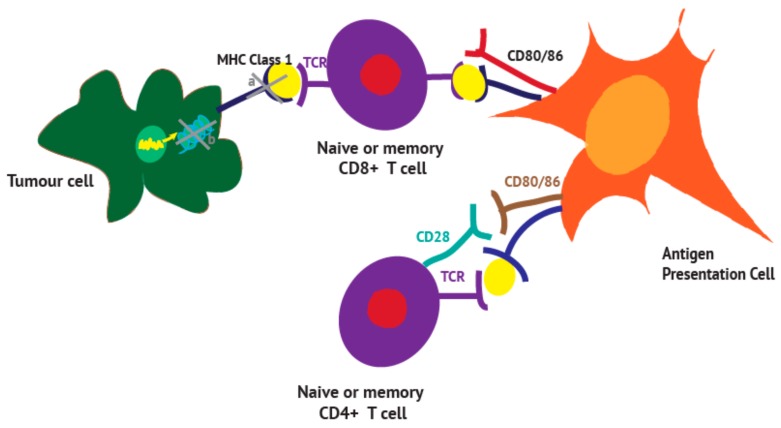
Key steps required for efficient priming of T cell responses within the cancer immunity cycle. Tumour antigen (yellow) is processed via the immune-proteasome (green) and other components of the antigen processing machinery leading to its expression on MHC class I molecule. Grey crosses indicate nodes at which tumour cells can lose antigen presentation rendering themselves ‘invisible’ to the immune system. Aside from loss of antigen expression itself, other ways that cancer cells can reduce their ability to present tumour antigen include: (a) alterations/loss of b2-microglobulin resulting in absence of MHC class I impairing target recognition by CD8^+^ T cells; and (b) impairments of the antigen processing machinery.

The concept that the body can differentiate between self and non-self tissue earned Macfarlane Burnett the Nobel Prize in 1960 [42] but a further half century of work was required to appreciate the complexity of how tumours coopt the immune system to ensure tolerance (Figure 1). Antigens released by dying cancer cells are ingested by dendritic cells and presented to CD8^+^ T-cells on MHC class I molecules. In order to induce a potent immune response, the antigen released must be accompanied by the emission of damage-associated molecular patterns (DAMPs) [43,44]. Surface-exposed DAMPs like heat-shock proteins (HSP 70/90), calreticulin (CRT) on cancer cells or secreted DAMPs such as adenosine triphopsphate (ATP), nucleic acids and high mobility group Table 1 protein (HMGB1) interact with respective receptors on DCs and lead to their maturation with upregulation of MHC class II expression [44,45]. Presentation of antigens by professional APC to naïve T cells requires at least 3 signals: (i) signal 1 which results from the interaction of the MHC/Ag complex with the T cell receptor (TCR) and sends an activating signal to the T cells, (ii) signal 2 which results from the interaction of the B7 molecules (CD80 and CD86) with the CD28 stimulatory receptor expressed on T cells and (iii) signal 3 which results from secretion of cytokines like IL-12 and interferon (INF) α/β from APC. Il-12 receptors are expressed on natural killer cells (NKs), B and T lymphocytes [46]. Binding of IL-12 leads to activation of the JAK-STAT (Janus kinases and signal transducer and activator of transcription proteins) pathway and thus to transcription of genes for immune cell activation. Il-12 also increases INF-γ production from NKs and T cells which in turn leads to increased antigen presentation through upregulation of MHC molecules [47]. The combination of these 3 signals is hence essential for the activation of CD4 (through MHC class II) and CD8 (through MHC class I) T cells. Priming CD4^+^ T-helper cells is necessary to generate effective CTL-mediated anti-tumour responses as well as long-lasting memory CTLs [16,44,48,49,50].

Loss or ineffective antigen presentation therefore both reduces direct antigen priming of naive T-cells and prevents the recognition of tumour cells by antigen-experienced T cells, thereby rendering tumour cells essentially ‘*invisible*’ to the immune system. Several mechanisms contribute to the defect of antigen presentation by tumour cells including firstly a lack of tumour antigens; a downregulation or loss of MHC expression, alterations of the machinery responsible for the loading of tumour antigens onto MHC or the loss of co-stimulatory molecules (Figure 1).

## 4. Cancer Vaccines

The idea of cancer vaccines was born more than 100 years ago [51]. Despite intensive effort and promising preclinical results, their implementation into clinic as therapeutic agents has been disappointing so far. However, the recent success of checkpoint inhibitors has paved the way for combination strategies and led to re-investigation of cancer vaccines. PROSPECT was a phase 3 randomized trial that investigated a PSA-targeted, poxvirus-based cancer vaccine alone or in combination with GM-CSF against placebo in patients with metastatic castration resistant prostate cancer. There was no significant difference in overall survival regarding the three different treatment arms (updated ASCO 2018) [52]. The PROSTVAC-V/F vaccine is now being investigated in combination with the checkpoint inhibitor nivolumab (NCT02933255). Table 2 gives an overview of different types of cancer vaccines that are currently being investigated in combination with checkpoint inhibitors. In general, there are two strategies for cancer vaccines—either direct delivery of the antigen into the patient (in vivo approach) or collecting monocytes from the patient followed by ex vivo antigen loading.

### 4.1. Ex Vivo Approaches

Dendritic cells (DCs) are the professional antigen-presenting cells of the immune system, capable of capturing exogenous antigens and not only presenting them in the MHC class II pathway but also presenting them in the class I antigen presentation pathway to CD8^+^ T cells (called ‘cross presentation’) which is essential for long lasting immune responses. The ability of DCs to cross present made them an ideal instrument for therapeutic use in the field of cancer immunotherapy. DC vaccines are generated ex vivo with the collection of monocytes from patients via leukapheresis which are then activated and differentiated in a laboratory, and subsequently loaded with whole tumour cells, DNA or mRNA encoding tumour antigens, or recombinant virus expressing tumour antigens as well as antigenic peptides or proteins [62,63,64,65]. The only FDA approved cancer vaccine, Sipuleucel-T, is based on this method. It is produced by ex vivo exposure of dendritic cell precursors to PA 2024, a fusion protein combining recombinant prostatic acid phosphatase with Granulocyte-macrophage colony-stimulating factor (GM-CSF). In the phase III IMPACT trial, Sipuleucel-T has shown an overall survival benefit of 4.1 months in men with castration-resistant prostate cancer when compared to placebo [34]. The IMPACT trial has not been left without criticism. First, the product itself is a mixture of monuclear cells, with less than 20% being antigen-presenting cells, so technically not a pure DC vaccine [62]. Secondly, no significant difference in biochemical failure or progression free survival could be shown. It is therefore hard to explain the difference in overall survival as effect of the treatment. There have been differences in the two treatment arms as two thirds of the cells harvested from the placebo group were not reinfused. This effect on its own could have potentially influenced the outcome [66,67]. 

Numerous trials utilising autologous DCs pulsed with autologous tumour RNA (NCT02993315, NCT01983748), synthetic mRNA encoding tumour antigens (e.g., TriMix) and synthetic peptides are ongoing across multiple tumour indications [23,68,69]. However, the optimal strategy for the strongest immune response to DC vaccines has not been identified yet. One of the challenges is the maturation process of DCs. Early trials have shown superiority for the delivery of mature DCs as immature DCs can potentially induce immunogenic tolerance against the used antigen [70,71,72]. Therefore, cytokine cocktails including tumour necrosis factor (TNF)-α or GM-CSF combinations with IL1ß, IL6, prostaglandin E2 and toll like receptor (TLR) agonists are used for DC maturation [63]. TNF-α and GM-CSF are needed to induce DC differentiation from monocytes or haematopoetic progenitor cells. PGE2 is thought to be mandatory for DC migration into lymph nodes [73]. However, there are data suggesting PGE2 reduces IL-12 production by DCs which is essential for T cell stimulation [73]. An option to overcome that problem is to add TLR agonists into the maturation cocktail. Upon activation TLRs recruit adaptor proteins which signal through NFκB and mitogen-activated protein kinase (MAPK). This induces transcription and translation of proinflammatory cytokines and MHC molecules [46]. Furthermore, different forms of vaccine administration, dosing and schedules exist and need to be compared in clinical trials to prove superiority of any of the protocols [74]. Currently under investigation are also different kinds of combination therapies involving administration of DC-based vaccines with chemotherapies, checkpoint inhibitors, TLR agonists and tyrosine kinase inhibitors (e.g., NCT02669719, NCT01697527, NCT02649829, NCT02678741, NCT03325101, NCT01876212, NCT01976585.

### 4.2. In Vivo Approaches

This strategy involves the direct delivery of the antigen (either nucleic acid-based or peptide based) into the patient, usually with the aid of an adjuvant where they are internalised and processed by antigen presenting cells. In contrast to ex vivo approaches, these can be produced fairly easily and induce minimal if any toxic effects. 

One of the key elements in creating peptide based vaccines is the length of the used peptides. *Short epitope peptides* are typically eight to ten amino-acids in length and directly bind to class I MHC molecules on the surface of immature DCs. However, as short peptides can also bind to class I MHC molecules on nonprofessional antigen-presenting cells (e.g., fibroblasts) which lack co-stimulatory molecules and thereby do not induce immune responses, this may induce immune tolerance [75,76]. *Long peptides* usually consist of 20–30 amino acids and require internalization by antigen presenting cells such as DCs.

Antigens alone are often poor inducers of immunity which can result in immune tolerance instead of immunity. Therefore, adjuvants are needed as vaccine components to enhance immune response [38,77]. The optimal vaccine adjuvant provides adequate availability of the antigen and enhances the immune response by inducing expression of co-stimulatory molecules and cytokines by APC. A widely used but controversially discussed adjuvant is the granulocyte-macrophage colony-stimulating factor (GM-CSF). It can be either delivered via secretion of viral-transduced tumour cells or as a recombinant protein given together with the vaccine intradermally or subcutaneously. Trials have shown varying success in achieving T cell responses, suggesting a dose-dependent immunosuppressive or immunostimulant effect of GM-CSF [78,79,80,81]. Other commonly used adjuvants are TLR agonists such as polyinosinic–polycytidylic acid stabilised with polylysine and carboxymethylcellulose (Poly-ICLC) that mimic microbial stimulation and thereby enhance T cell responses. Aluminium salts induce a local inflammatory response that results in trafficking of APC to the injection site [38,77]. More recently, pre-conditioning the vaccine site with tetanus-diphtheria toxoid has shown improved lymph node homing of DCs in a phase 2 trial with glioblastoma patients. Also, the delivery process of antigens influences the efficacy of the cancer vaccine. Micro/nanoparticles can protect the antigen from degradation and are also useful for antigen trafficking to desired organs such as lymph nodes or the spleen. Liposomes, synthetic polymers or lipoprotein nanodiscs have been used for antigen delivery and allow for combining antigen with cytokines such as interleukin-2 and GM-CSF into a single particle [16,82].

Targeting *tumour associated antigens* with peptide-based cancer vaccines has been a long time focus of industry as they are shared by several cancer types. However, their use in clinical practice has been hindered so far by disappointing phase 3 clinical trials [26,37,83,84,85,86,87,88,89]. More promising results could be shown in patients with human papilloma virus type 16 (HPV-16) positive non-invasive vulvar lesions. Therapeutic vaccination with HPV-16 E6 and E7 synthetic long peptides led to clinical responses in a phase II study in 12 out of 20 patients, showing complete responses of lesions in five patients. The grade of clinical response was associated with the strength of vaccine-induced T-cell response [21,90,91]. Ongoing trials in genital cancers are currently investigating combination modalities with HPV vaccines and topicals like imiquimod or fluorouracil (NCT03196180, NCT00788164).

## 5. Personalised Cancer Vaccines

With the establishment of next generation sequencing (NGS) for detection of tumour mutations the idea of generating vaccines that target personal tumour neoantigens was born.

Non-synonymous somatic mutations are identified by whole exome sequencing of tumours and normal-cell DNA from individual patients. The listed mutations are then ranked according to their likelihood of expression and affinity binding of the neoantigen to autologous MHC class I and II molecules which can be predicted by bioinformatic tools like NetMHCpan or IEDB [25,59].

The advantage of neo-antigens that arise from tumour specific mutation is that they are highly immunogenic since the cytotoxic T lymphocyte clones with high affinity for these antigens are unlikely to have been deleted by central tolerance. This concept has been investigated in a phase 1 study with six stage III and IV melanoma patients after surgical resection with curative intent. These patients received subcutaneous vaccinations of synthesized long peptides targeting up to 20 personal neo-antigens per patient, combined with the TLR 3 and melanoma differentiation associated protein 5 agonist poly-ICLC as immunostimulant. After a median follow-up of 25 months four patients remained without disease-recurrence [25]. A currently ongoing phase 1 study is investigating a mutation derived-based personalized vaccine in glioblastoma patients. The vaccine consists of several peptides based on each patient’s own tumour sequence. The vaccine is given after radiation and chemotherapy, in the maintenance phase of temozolomide and tumour treating fields (NCT03223103). Another phase 1 study is also using the concept of a personalized peptide vaccine in patients with advanced pancreatic adenocarcinoma or colorectal adenocarcinoma in combination with the checkpoint-inhibitor pembrolizumab (NCT02600949). The most recent technology is the use of mRNA-based cancer vaccines [82]. The IVAC-Mutanome study was a phase 1 study including 13 melanoma with advanced disease (stage III and IV) [59]. Ten mutations per patients were selected and two synthetic RNA molecules coding for five (27 mer) peptides with the mutation in position 14 were synthesized in vitro. The RNA molecules were then linked to a MHC trafficking signal peptide for optimized routing and presentation to MHC class I and II presentation. Patients received eight ultrasound-guided injections into inguinal lymph nodes. Immunogenicity was analysed by IFNγ-ELISpot in CD4^+^ and CD8^+^ T cells in pre and post-vaccination leukapheresis samples. Responses against one-fifth of the mutations were detectable in blood without in-vitro stimulation. Two of the five patients with metastatic disease experienced vaccine-related objective responses. Removal of lymph node metastasis in one patient confirmed vaccine-induced neo-epitope specific T cells in the tumour [59]. 

A currently ongoing phase 1 study is investigating an intravenous formulation of a RNA based personalized vaccine in combination with the PD-L1 targeting agent atezolizumab in patients with solid tumours (NCT03289962).

## 6. Challenges

### 6.1. Choosing the Right Antigen—Improving Bioinformatics

With the possibility of parallel sequencing, a new era for antigen selection began. More challenging is identifying which of the listed mutations will induce the strongest immunogenicity in vivo [92]. Bioinformatic prediction tools try to rank the immunogenicity of the antigen according to the binding affinity of the predicted epitope to individual MHC molecules, the likelihood of presentation, the clonality and the level of expression of the associated RNA. However, recent trials have shown that CD8^+^ responses against predicted high-affinity binders were low as 29%, indicating the need for improvement of the used algorithms [59]. 

### 6.2. Choosing the Right Combination

As cancer cells have evolved various mechanisms for immune escape, combination therapies are needed to restore antitumour-immunity. Conventional therapies like chemotherapy and radiotherapy can be used to support antigen release by cancer cell death. Checkpoint inhibitors release the break on endogenous T cells by blocking the negative regulatory pathway used by tumours. They have shown efficacy on their own in various cancer types, however, less success was achieved in tumours devoid of infiltrating lymphocytes [14]. The lack of infiltrating T cells might be the result of a tumour suppressive microenvironment created by the cancer cells through the release of immunosuppressive cytokines, recruitment of regulatory T cells and myeloid-derived suppressor cells. A high expression of active indoleamine-pyrole 2,3-dioxygenase (IDO) in cancer cells leads to immunosuppression by depletion of tryptophan which results in promotion of T regulator cells [86,93,94]. For the migration of T cells through the vascular endothelium at the tumour site expression of intercellular adhesion molecules (ICAM-1) and vascular adhesion molecules (VCAM-1) are needed. Angiogenic molecules, like the vascular endothelial growth factor (VEGF), at the tumour microenvironment inhibit expression of endothelial adhesion molecules and thereby T cell migration [95,96]. Combination therapies with VEGF inhibitors, TGF-ß inhibitors or newer immunomodulators like IDO inhibitors might be helpful to overcome the tumour-suppressive microenvironment and are currently under investigation in clinical trials (NCT02873962, NCT02423343, NCT03347123) [93]. Depletion of Tregs can also be achieved by conventional chemotherapy like cyclophosphamide.

### 6.3. Choosing the Right Time—Adjuvant vs. Palliative?

Tumour immunosuppression often correlates with tumour burden, making immunotherapy less effective in patients with advanced disease. In clinical trials immunologic response rates to vaccines are often higher during adjuvant treatment than in the palliative setting which provides a rationale for the use of vaccines in earlier stage of disease [59,97,98]. Furthermore, current approaches of personalized vaccines are technically challenging and manufacturing time of several months might be challenging for patients with advanced disease. Again, combination strategies could be used to bridge the time between vaccine manufacturing and application.

### 6.4. Tumour Evolution and Loss of Antigen

With tumour progression new mutations emerge which can lead to ineffectiveness of neo-epitope vaccines because of mutation and loss of the antigenicity of the neoepitope itself [25,59]. For target recognition, T cells depend on antigen processing and presentation through MHC proteins. Downregulation of MHC class I proteins on the cancer results in reduced antigen presentation and thus facilitates immune evasion. The down regulation of MHC class I proteins has been observed in various cancer types [99,100,101,102,103,104]. It can happen either on the genetic level (mutation or deletion of the MHC class I gene) or be the result of a defect in protein generation [105]. In order for antigens to bind to MHC class I they are typically cleaved into peptide fragments by immunoproteasome in the cytosol of cells. The proteasome complex is a multi-catalytic enzyme complex and down-regulation of subunits of the proteasome complex has been associated with tumour growth and metastases. The linkage of the small peptide fragments to MHC class I takes place in the endoplasmatic reticulum. A defect of the transporter associated with antigen processing (TAP) at the endoplasmatic reticulum or a loss of the endoplasmatic aminopeptidases (ERAP 1 and ERAP 2) can result in a further reduction of antigen expression [106].

## 7. Conclusions

The acceleration of NGS–omics technologies together with rapid progresses in bioinformatics heralds a very exciting era for research in oncology. There is abundant data detailing how a tumour is different from normal self tissue on a DNA, RNA and protein level. This does not only help the search for targeted therapies but also provides possibilities for personalized treatment approaches. Further development of techniques for collecting cell free DNA or circulating tumour cells are also likely to provide a more ‘up to date’ picture of the cancer, enabling approaches to overcome the problem of tumour heterogeneity and cancer evolution. Analysis of the tumour microenvironment and understanding strategies used by cancer to overcome immune evasion will also open the possibilities of successful combinatorial therapies.

## Figures and Tables

**Table 1:** Examples of tumour antigens and vaccine trials.

Type of Tumour Antigen		Examples (Ref.)
Tumour associated	Overexpression	HER2 [32] TERT [33]
	Tissue differentiation	PSA [34] Mammaglobin-A [35] Tyrosinase [36]
	Cancer-germline	MAGE [37] BAGE [38] NY-ESO-1 [26]
	Oncofetal	CEA [39] MUC-1 [39] TPBG [16]
Tumour specific	Oncogenic viral	HPV [21] HBV [20] HHV-8 [30]
	Neoantigens	BRAF V600E [40] KRAS G12D [41]

**Table 2:** Examples of clinical trials combining different type of cancer vaccines with checkpoint inhibitors. The result section gives an overview of results from earlier phase trials if data are available for the specific cancer type. Abbreviations: TNBC, triple negative breast cancer; NSCLC, non-small cell lung cancer; SCLC, small cell lung cancer; OS, overall survival; PFS, progression free survival; WT 1, Wilms tumour gene 1; id, intradermal; im, intramuscular; sc, subcutaneous; iv, intravenous.

Clinical Trials.gov Identifier; Phase	Tumour Type; Setting	Intervention	Mode of Action	Results from Previous Vaccine Trials
NCT03328026; Phase 1/2	Breast cancer; palliative	SV-BR-1-GM id., pembrolizumab, ipilimumab, cyclophosphamide, interferon	GM-CSF secreting whole cell vaccine, anti-PD-1, anti-CTLA-4, chemotherapy, cytokine	Clinical responses seen in monotherapy with SV-BR-1-GM (phase 1 NCT03066947)
NCT02826434; Phase 1	TNBC; adjuvant	PVX-410 im., durvalumab	Peptide vaccine, anti-PD-L1	–
NCT03199040; Phase 1	TNBC; adjuvant	Neoantigen DNA vaccine im. alone or plus durvalumab	DNA vaccine, anti-PD-L1	–
NCT03362060; Phase 1	TNBC; palliative	PVX-410 sc. alone or plus pembrolizumab	Peptide vaccine, anti-PD-1	–
NCT02451982; Phase 1/2	Pancreatic cancer; neoadjuvant and adjuvant	GVAX id., cyclophosphamide alone or plus nivolumab	GM-CSF secreting whole tumour cell vaccine, chemotherapy, anti-PD-1	Palliative phase 2 study (ECLIPSE) with GVAX and cyclophosphamide and CRS-207 showed no OS compared to standard of care (ASCO GI abstract 2017)
NCT03050814; Phase 2	Colorectal cancer; palliative	Standard of care alone or plus ad-CEA vaccine sc. and avelumab	Adenovirus vector vaccine expressing CEA, anti-PD-L1	Ad-CEA induced T cell mediated immune response measured by IFNγ Elispot (phase 1) [53]
NCT03152565; Phase 1/2	Colorectal cancer; palliative	ADC id., avelumab	Autologous dendritic cell vaccine, anti-PD-L1	–
NCT03029403; Phase 2	Ovarian, tubal, peritoneal; palliative	DPX survivac sc., cyclophosphamide, pembrolizumab	Survivin targeting peptide vaccine, chemotherapy, anti-PD-1	DPX induced CD8^+^ T-cell responses, measured by IFNγ Elispot (phase 1) [54]
NCT02499835; Phase 1	Prostate cancer; palliative	pTVG-HP id., pembrolizumab	Plasmid DNA vaccine encoding prostatic acid phosphatase, anti-PD-1	pTVG-HP induced CD8^+^ T-cell responses, measured by IFNγ Elispot (phase 1) [55]
NCT02933255; Phase 1/2	Prostate cancer; metastatic and localized	PROSTVAC sc., nivolumab	Poxvirus expressing PSA vaccine, anti-PD-1	PROSTVAC alone no difference in OS (phase 3, ASCO abstract 2018)
NCT02808143; Phase 1	Non-muscle-invasive bladder cancer; recurrent	BCG, pembrolizumab intravesically	BCG, anti-PD-1	–
NCT03164772; Phase 1/2	NSCLC; palliative	BI 1361849 id., durvalumab alone or plus tremelimumab	mRNA vaccine, anti-PDL-1, anti-CTLA-4	–
NCT02879760; Phase 1/2	NSCLC; palliative	Ad-MAGEA3 im., MG1-MAGEA3 iv., pembrolizumab	Adenovirus vaccine expressing MAGEA3, Maraba virus expressing MAGEA3, anti-PD-1	–
NCT03380871; Phase 1	NSCLC; palliative	NEO-PV-01 sc., pembrolizumab, carboplatin, pemetrexed	Personalized cancer vaccine, anti-PD-1, chemotherapy	–
NCT02955290; Phase 1/2	NSCLC; palliative	CIMAvax im., nivolumab	Peptide vaccine containing recombinant human EGF, anti-PD-1	Phase 2 study of CIMAvax showed increased OS for patients with good anti-EGF antibody response [56]
NCT02823990; Phase 2	NSCLC; palliative	TG4010 sc., nivolumab	Ankara-virus vaccine expressing MUC1and IL-2, anti-PD-1	Phase 2 of first line chemo with TG4010 or placebo showed improved PFS for the vaccine arm [57]
NCT02439450; Phase 1/2	NSCLC; palliative	Viagenpumatucel-L id., nivolumab	gp96-Ig secreting lung cancer cells, anti-PD-1	–
NCT03406715; Phase 2	SCLC; palliative	Ad.p53-DC id., nivolumab, ipilimumab	Autologous dendritc cell based p53 vaccine, anti-PD-1, anti-CTLA-4	Ad.p53-DC induced immune-cell responses, measured by IFNγ Elispot (phase 1) [58]
NCT02775292; Phase 1	Solid tumours; palliative	NY-ESO-1 TCR iv., NY-ESO-1 DC id., nivolumab, cyclophosphamide, fludarabine	Gene modified T cells, peptide-pulsed dendritic cells, anti-PD-1, chemotherapy	–
NCT03289962; Phase 1	Solid tumours; palliative	RO7198457 iv., atezolizumab	Personalized RNA mutanome vaccine, anti-PD-L1	RO7198457 induced T cell mediated immune response measured by IFNγ Elispot (phase 1) [59]
NCT03311334; Phase 1	Solid tumours; palliative	DSP-7888 id., nivolumab or atezolizumab	WT1 protein-derived peptide vaccine, anti-PD-1 or anti-PD-L1	–
NCT03162224; Phase 1/2	Head and neck cancer; palliative	MEDI0457 im., durvalumab	HPV DNA vaccine, anti-PD-L1	–
NCT03260023; Phase 1/2	HPV-16 positive cancer; palliative	TG4001 sc., avelumab	Modified vaccinia of Ankara-virus expressing HPV 16 and IL-2, anti PD-L1	Clinical responses seen in patients with HPV-16 related cervical intraepithelial neoplasia after TG4001 injections (phase 2) [60]
NCT03047928; Phase 1/2	Melanoma; palliative	PD-L1/IDO vaccine sc., nivolumab	Peptide based vaccine, anti-PD-1	–
NCT02385669; Phase 1/2	Melanoma; neoadjuvant, adjuvant, palliative	6MHP, ipilimumab	Melanoma-associated helper peptide vaccine, anti-CTLA-4	6MHP decreased CD8^+^ T-cell responses, measured by IFNγ Elispot (phase 1) [61]
